# MicroRNA-124/Death-Associated Protein Kinase 1 Signaling Regulates Neuronal Apoptosis in Traumatic Brain Injury *via* Phosphorylating NR2B

**DOI:** 10.3389/fncel.2022.892197

**Published:** 2022-06-15

**Authors:** Yingwu Shi, Wenxing Cui, Qiang Wang, Jinpeng Zhou, Xun Wu, Jin Wang, Shenghao Zhang, Qing Hu, Liying Han, Yong Du, Shunnan Ge, Haixiao Liu, Yan Qu

**Affiliations:** Department of Neurosurgery, Tangdu Hospital, Fourth Military Medical University, Xi’an, China

**Keywords:** traumatic brain injury, DAPK1, miR-124, NR2B, neuronal apoptosis

## Abstract

Death-associated protein kinase 1 (DAPK1), a Ca^2+^/calmodulin-dependent serine/threonine-protein kinase, promotes neurons apoptosis in ischemic stroke and Alzheimer’s disease (AD). We hypothesized that knockdown DAPK1 may play a protective role in traumatic brain injury (TBI) and explore underlying molecular mechanisms. ELISA, Western blotting, immunofluorescence, dual-luciferase assay, and Reverse Transcription and quantitative Polymerase Chain Reaction (RT-qPCR) were used to determine the mechanism for the role of DAPK1 in TBI. Open field and novel objective recognition tests examined motor and memory functions. The morphology and number of synapses were observed by transmission electron microscopy and Golgi staining. DAPK1 was mainly found in neurons and significantly increased in TBI patients and TBI mice. The dual-luciferase assay showed that DAPK1 was upregulated by miR-124 loss. The number of TUNEL^+^ cells, expression levels of cleaved caspase3 and p-NR2B/NR2B were significantly reduced after knocking-down DAPK1 or overexpressing miR-124 in TBI mice; and motor and memory dysfunction was recovered. After Tat-NR2B were injected in TBI mice, pathological and behavioral changes were mitigated while the morphology while the number of synapses were not affected. Overall, DAPK1 is a downstream target gene of miR-124 that regulates neuronal apoptosis in TBI mice *via* NR2B. What’s more, DAPK1 restores motor and memory dysfunctions without affecting the number and morphology of synapses.

## Introduction

Traumatic brain injury (TBI) is a high disability and fatality disease (Jiang et al., 2019). Approximately 43% of patients with TBI experienced long-term dysfunctions because of overactivated neuronal apoptosis and the non-renewability of neurons (Su et al., 2019; Capizzi et al., 2020). Death-associated protein kinase 1 (DAPK1) is a Ca^2+^/calmodulin-dependent serine/threonine-protein kinase, which promotes neuronal apoptosis in neurons during various neurological disorders such as ischemic stroke and Alzheimer’s disease (AD) (Shu et al., 2016; Kim et al., 2019; Wang et al., 2020). DAPK1 leads to neuronal apoptosis in ischemic stroke *via* phosphorylating NR2B (p-NR2B), P53, or Tau (Tu et al., 2010; Pei et al., 2015; Wang et al., 2017). In AD, activated DAPK1/NDRG2 pathways lead to cell death, and DAPK1 triggers the amyloidogenic pathway and Aβ production by phosphorylating APP (Kim et al., 2016; Xu et al., 2019). However, it is unclear whether DAPK1 contributes to TBI.

NR2B is a subunit of N-methyl-D-aspartic acid (NMDA) receptors and regulates Ca^2+^ influx in neurons (Brown et al., 2019), which helps maintain of normal cellular functions and neuronal plasticity (Wang et al., 2014); previous evidence demonstrating that inhibiting the NR2B phosphorylation can rescue TBI-induced neurological impairment (Schumann et al., 2008). As showed in the results of this work, the NR2B level was not changed in TBI group nor by any of the rescue strategies. In cerebral ischemia, the phosphorylation of NR2B can cause neuronal apoptosis by promoting excessive Ca^2+^ influx, the downregulation of cAMP-response element binding protein (CREB), the activation of JUN, neuronal nitric oxide synthase (nNOS) and post-synaptic density protein 95 (PSD95) (Sun et al., 2015; Dai et al., 2016). PSD95 is an abundant scaffolding protein located at excitatory synapses, which specifically binds to NR2B through its PDZ domains. The NR2B–PSD95 complex interaction with activated calmodulin-dependent protein kinase II (CaMK II), resulting in excessive Ca^2+^ influx (Irie et al., 1997). However, the interaction between DAPK1 and NR2B in TBI remains unknown.

Recent research has focused on non-coding RNA, particularly miRNA, as it plays a vital role in gene post-transcriptional regulation and RNA silencing (Bushati and Cohen, 2007; Barry, 2014). The role of miR-124 has been reported in various neurological diseases. In AD, miR-124 can mediate synaptic and memory deficits through Potential Enhancers of Cancer Immunotherapy and Type 1 (PTPN1) (Wang et al., 2018). Besides, the early miR-124 treatment-induced neuroprotection and functional improvement in the case of focal cerebral ischemia stroke (Yang et al., 2017). In Parkinson’s disease (PD), overexpression of miR-124 could effectively inhibit DAPK1 expressions and alleviate MPP^+^ induced cell apoptosis (Lu et al., 2020), while the interaction between miR-124 and NR2B has not been reported. Previous research found that miR-124 can promote M2 polarization of microglia and increase hippocampus neurogenesis by inhibiting Toll-like receptor 4 (TLR4) (Yang et al., 2019). These findings suggest that miR-124 can be a promising target for TBI therapy.

This study firstly showed that DAPK1 was upregulated in TBI patients and TBI mice due to miR-124 loss. Overexpression of miR-124 or knocking down DAPK1 can rescue TBI-mediated behavioral and pathological changes. Finally, Tat-NR2B that can block the binding between DAPK1 and NR2B was used in TBI mice, and the results indicate that TBI-induced dysfunction was mitigated while normal synapse function was not affected.

## Materials and Methods

### Patients

This study was approved by the Ethics Committee of Tangdu Hospital, Fourth Military Medical University, and was conducted from September 2020 to April 2021. Patients were between the ages of 18 and 80 diagnosed with TBI *via* head computerized tomography (CT) findings at admission were included in this study. Patients with a history of neurological disease or severe systemic disease (uremia, cirrhosis, or malignant cancer) were excluded from the study. Using 6-month Glasgow Outcome Scale (GOS) score to evaluate the neurologic function. GOS scores of 1∼3 were unfavorable outcomes, and scores of 4–5 were considered favorable outcomes. Preoperative medical records were searched for age, gender, pupil reaction, mechanism of injury, and laboratory biochemical examinations. The hematoma volume on admission CT scan was measured by ABC/2 method as reported before (Kothari et al., 1996). All experimental procedure conformed to the Declaration of Helsiniki.

### ELISAs

Cubital venous blood samples were collected from TBI patients who were injured within 24 h and met the inclusion/exclusion criteria. Hemolytic samples were excluded from this study. Plasma was obtained by centrifuging of whole blood at 1,500 g for 15 min. An ELISA kit (Jianglaibio, China) was used to measure the expression levels of DAPK1 in the plasma, and all procedures were completed as per the manufacturer’s instructions.

### Animals

The Fourth Military Medical University’s Ethics Committee approved all experimental procedures. All experiments were performed in accordance with the National Institutes of Health Guide for the Care and Use of Laboratory Animals. Eight weeks old, male C57BL/6 mice were purchased from the Animal Center of the Fourth Military Medical University. All animals were housed in specific pathogen-free environment room (*ad libitum* to food and water) with a 12-h light/dark cycle. The experimental holding room had a temperature (23°C) and humidity control (60%).

### Controlled Cortical Impact Model of Traumatic Brain Injury

Mice were subjected to either controlled cortical impact (CCI) or sham surgery, as previously described (Shi et al., 2022). First, the mice were deeply anesthetized with isoflurane, and their heads were fixed on a stereotactic device (RWD, China). The position of craniotomy was the right parietal bone window (1.5 mm from the midline and 1.5 mm behind the bregma) and a 2.0 mm diameter dental drill was used. Then, the skull cap was carefully removed while not damaging the underlying leptomeninges. The cortex was struck with a flat metal tip at a speed of 3.0 m/s and a depth of 1.8 mm; the contact time was 2.0 s. Each mouse underwent with single strikes. After CCI the window on the skull was sealed with bone wax, and the incision was sutured. The mice were allowed to recover on a heating pad to maintain their core body temperature at 37°C. A similar surgical procedure was performed on sham mice but without CCI.

### Immunofluorescence Staining

The mice were deeply anesthetized with isoflurane and transcardially perfused with 30 ml of 0.01 M PBS followed by 60 ml of 4%(w/v) formaldehyde 7 days after TBI. Then mice brains were removed quickly and postfixed with 4% (w/v) formaldehyde overnight at 4°C. After dehydrating in 10, 20, and 30% (w/v) sucrose solutions, the brains were cut into 30 μm frontal sections. The sections were incubated with 0.01 M PBS containing 0.1% (v/v) Triton X-100 for 10 min and then blocked with 0.01 M PBS containing 5% (w/v) goat serum for 1 h. The sections were incubated for 12 h at room temperature with the following antibodies: Chicken anti-GFAP (1:300; Invitrogen, California, CA, United States), goat anti-Iba1 (1:300; Abcam, Cambridge, United Kingdom), guineapig anti-NeuN antibody (1:300; Millipore, Massachusetts, MA, United States), rabbit anti-DAPK1 (1:300; Cell Signaling Technology, Massachusetts, MA, United States). Then the sections were washed and laster incubated for 1 h at room temperature with 594 anti-goat (1:500; Invitrogen, California, CA, United States), 594 anti-chicken (1:500; Invitrogen, California, CA, United States), 594 anti-guineapig (1:500; Invitrogen, California, CA, United States), or 488 anti-rabbit (1:500; Invitrogen, California, CA, United States). Finally, the sections were dyed in DAPI solution (1 mg/ml) for 10 min before images were captured on a confocal microscope (A1, Nikon, Tokyo, Japan).

### TUNEL Staining

Cell apoptosis was measured by *In Situ* Cell Death Detection Kit (Roche, Basel, Switzerland). Briefly, the sections were incubated with 0.01 M PBS containing 0.1% (v/v) Triton X-100 for 10 min. Then the sections were dyed using the TUNEL reaction solution prepared in a humidified dark box for 1 h at room temperature. Finally, the tissues were dyed in DAPI solution (1 μg/ml) for 10 min before images were captured on a confocal microscope (Nikon, Tokyo, Japan).

### Western Blotting

The perilesional cortex was collected under a dissecting microscope at 7 days after TBI and extracted in RIPA lysis buffer containing 1% (v/v) protease and phosphatase inhibitor cocktail. The protein samples were separated by SDS-PAGE and transferred to PVDF. After being blocked with 5% (w/v) non-fat milk, the membranes were incubated overnight at 4°C with the following primary antibodies: Rabbit anti-Cleaved caspase 3 (1:1,000, Cell Signaling Technology, United States), Rabbit anti-DAPK1 (1:1,000, Cell Signaling Technology, Massachusetts, MA, United States), Rabbit anti-β-actin (1:1,000, Abcam, Cambridge, United Kingdom), Rabbit anti-NR2B (1:1,000, Abcam, Cambridge, United Kingdom), Rabbit anti-p-NR2B (Ser1303, 1:1,000, Abcam, Cambridge, United Kingdom), Rabbit anti-PSD95 (1:1,000, Proteintech, China), Rabbit anti-ERK1/2 (1:1,000, Cell Signaling Technology, Massachusetts, MA, United States), Rabbit anti-p-ERK1/2 (Thr202/Tyr204, 1:1,000, Cell Signaling Technology, Massachusetts, MA, United States). Afterward, the membranes were incubated with HRP anti-rabbit (1:1,000, Cell Signaling Technology, Massachusetts, MA, United States) for 1 h at room temperature and then scanned with a Bio-Rad (California, United States) gel imaging system.

### Dual-Luciferase Reporter Assay

A wild-type and a mutant DAPK1 3′ UTR were cloned into the pSI-Check2 renilla luciferase reporter plasmid (Hanbio Biotechnology, China). HEK (Human embryonic kidney) 293T cells were cotransfected with the negative control (NC) mimics or miR-124 and wild-type (WT) or mutant (Mut) DAPK1 3′ UTR plasmid. The cells were harvested, and cell lysates were assayed for firefly and renilla luciferase activities using the dual-luciferase reporter assay system (Promega, Wisconsin, WI, Untied States). The normalized values (Renilla luciferase/firefly activity) were used for analysis.

### Reverse Transcription and Quantitative Polymerase Chain Reaction

Seven days after TBI, total RNA of the cortex was extracted using TRIzol Reagent (Invitrogen, California, CA, United States) according to the manufacturer’s protocol. According to the manufacturer’s instructions, the miscript cDNA synthesis kit (Tiangen, China) was used for the reverse transcription reaction. Using miRNA isolation kit (Tiangen, China) to extract miRNA, RT–qPCR was performed using an iQ™ 5 Optical Module Real-Time PCR Detection (Bio-rad, California, CA, United States). ChamQ™ SYBR qPCR master mix was used to quantify miRNA, according to manufacturer’s instructions. Using the relative CT method to compare different samples. The fold increase or decrease was determined relative to a vehicle-treated control after normalizing to a housekeeping gene using 2^–Δ^ ^Δ^ *^CT^*. The primers used are listed in Supplementary Table 1.

### Delivery of Adeno-Associated Viruses

Injection of AAV2-hsyn-shDAPK1-eGFP to knock down DAPK1 or Con AAV (AAV2-hsyn -eGFP) into the right cortex area, and the viral titer was 1.0 × 10^9^ particles/ml. The stereotaxic coordinates for the cortex injection were anterior-posterior 1.80 mm, medial-lateral 2.50 mm, and dorsal-ventral 1.00 mm. The volume of injected virus was 2.0 μl and injection rate was 0.2 μl/min. After 2 weeks, the mice were subjected to either a sham procedure or CCI, followed by behavioral and pathological tests 1 week later.

### Intranasal Delivery of Agomir

Two weeks before TBI induction, the mice were grasped from the back every day, and kept their head and abdomen upward (Hanson et al., 2013). The mouse was fixed in this position for 1 min and then administered agomir in 4 μl drops by pipette, alternating between each nostril every 1 min. The mice were administered different doses (0.01, 0.05, 0.10, 0.50, 1.00, and 5.00 nmol) of agomir (sense sequence: 5′-UAAGGCACGCGGUGAAUGCC-3′, antisense sequence: 5′-CAUUCACCGCGUGCCUUAUU-3′) (GenePharma, China) by nasal instillation daily for 7 days after TBI induction. Control mice received an equal volume of solvent.

### Open Field Test

The open field chamber was 43.2 × 43.2 cm with opaque walls (30.5 cm). Seven days after TBI, mice were placed into the center of the apparatus individually and allowed to travel freely for 10 min. Locomotor activity and total distance traveled was recorded using an overhead camera connected to a computer with video tracking software (Noldus Ethovision version 8.0, Wageningen, Netherlands).

### Novel Objective Recognition Test

On day 6 after TBI, mice were individually placed in the same open field as described before for 10 min for habituation. On day 7 and 8 (training days) after TBI two identical cylinders (a height of 8 cm and diameter of 4 cm, made of polyethylene) were fixed at one side of open field and 10 cm from both side walls; mice were placed at the center of open field with their backs to two objects and allowed to travel freely for 10 min; their locomotor activity was recorded using an overhead camera linked to a computer running video tracking software (Noldus Ethovision version 8.0, Wageningen, Netherlands). On day 9 (testing day) after TBI, replace one of the cylinders with a cone (a height of 8 cm and diameter of 4 cm, made of polyethylene), repeat the above steps. Object recognizing was defined when the distance between the nose and the object was less than 2 cm (Leger et al., 2013). Time exploring around the objects was measured. Recognition index = (time exploring the novel object)/(time exploring the novel object + time exploring the familiar object) × 100%.

### Quantify of Lesion Volume

Seven days after TBI, A 3T small-animal Magnetic Resonance Imaging (MRI) scanner (UMR780, United imaging, China) was used to generate series of brain images. T2-weighted imaging (T2) was performed to assess total lesion volume. The setup parameters were as follows: repetition time (TR) = 1,000 ms, echo time (TE) = 110.5 ms, field of view (FOV) = 90 × 90 mm^2^, image matrix = 336 × 446, and 0.7-mm slice thickness. Image J (Version 1.53C, Maryland, MD, United States) was used to calculate the lesion volume in each brain.

### Administration of Peptides

After TBI induction, the mice were intravenously administered 10 mg/kg Tat-NR2B (YGRKKRRQRRR-KKNRNKLRRQHSY) or the scramble control peptide (Tat-sNR2B, YGRKKRRQRRR-NRRRNSKLQHKKY) once every day for 7 days. The peptides with 99% purity were synthesized by Sangon Biotech (Shanghai, China).

### Transmission Electron Microscope

On the seventh day after TBI, the mice were deeply anesthetized and perfused as before. Cortical brain regions were trimmed into 1 mm wide blocks in ice-cold PBS. After fixation overnight in 4% (v/v) glutaraldehyde and then in 1% (w/v) osmium tetroxide for 1 h, the brain sections were dehydrated in a graded ethanol immersion series and embedded in resin. Brain tissue pieces were cut into 80 nm sections using an ultramicrotome (Leica, Wetzlar, Germany). The micrographs were captured using a charge-coupled device camera (HT7700, HITACHI, Hitachi, Japan).

### Golgi Staining

Seven days after TBI, the mice were perfused as before, and the brains were sliced in 150 μm and then incubated in Golgi-Cox solution at room temperature for 5 days. The bleach-section staining was carried out as follows: rinsing with distilled water for 2 × 5 min, dehydrating with 50% (v/v) ethanol for 5 min, incubating with 3:1 (v/v) ratio ammonia for 10 min, incubating with 5% (w/v) sodium thiosulfate for 10 min (light forbidden), dehydrating with gradient ethanol, clearing with xylene for 2 × 10 min, and finally mounting with resinene.

### Statistical Analysis

SPSS 21.0 (IBM, New York, NY, United States) and Prism 8 (Graphpad, California, CA, Untied States) were used for statistical analysis and graphing. A two-tailed t-test was used for comparisons between two groups. One-way analysis of variance (ANOVA) was used for comparisons between more than two groups. For comparisons between two independent variables, Two-way ANOVA was used. For dichotomous variables, χ^2^ statistics were performed. Data are presented as mean ± SEM. *p* < 0.05 was considered statistically significant.

## Results

### Elevated Death-Associated Protein Kinase 1 Expression in Traumatic Brain Injury Patient Plasma Correlated With Poor Prognosis

This study included 147 TBI patients, with 46 (31.3%) having an unfavorable outcome. The univariate analysis results showed age, GCS score, abnormal pupil reaction, tracheotomy, abnormal AST, hyperglycemia and the expression of DAPK1 in plasma were significantly correlated with TBI patient’s outcome. The expression of DAPK1 in plasma of TBI patients with favorable outcome and unfavorable outcome were 483.99 ± 250.60 pg/ml and 731.58 ± 273.15 pg/ml, respectively. Furthermore, the multivariate analysis revealed that a high level of DAPK1 expression in plasma is an independent risk factor for an unfavorable TBI outcome (Table 1).

**TABLE 1:** Characteristics of the study population.

	Favorable outcome (*n* = 101)	Unfavorable outcome (*n* = 46)	Univariate analysis (*p*-value)	Multivariate analysis (*p*-value)
Age	47 (34, 56)	55 (47, 65)	<0.01	0.044
Male	79 (78.2%)	35 (76.1%)	0.774	
GCS score	11 (7, 13)	9 (8, 10)	<0.01	<0.01
Abnormal pupil reaction	50 (49.5%)	36 (78.3%)	<0.01	0.022
Marshall CT grade >II	70 (69.3%)	34 (73.9%)	0.569	
Tracheotomy	31 (30.7%)	27 (58.7%)	<0.01	
Mechanism of injury			0.247	
Motor vehicle	56 (55.4%)	31 (67.4%)		
Fall	23 (22.8%)	8 (17.4%)		
Strike	8 (7.9%)	5 (10.9%)		
Others	14 (13.9%)	2 (4.3%)		
**Laboratory biochemical examinations**				
Abnormal RBC	60 (59.4%)	22 (47.8%)	0.190	
Abnormal ALT	46 (45.5%)	28 (60.9%)	0.085	
Abnormal AST	41 (40.6%)	27 (58.7%)	0.041	0.023
Hyperglycaemia	42 (41.6%)	33 (71.7%)	<0.01	<0.01
Coagulopathy	34 (33.7%)	19 (41.3%)	0.371	
DAPK1 levels in plasma	483.99 ±250.60	731.58 ±273.15	<0.01	<0.01

### Upregulation of Death-Associated Protein Kinase 1 in Mouse Brain After Traumatic Brain Injury

**FIGURE 1:**
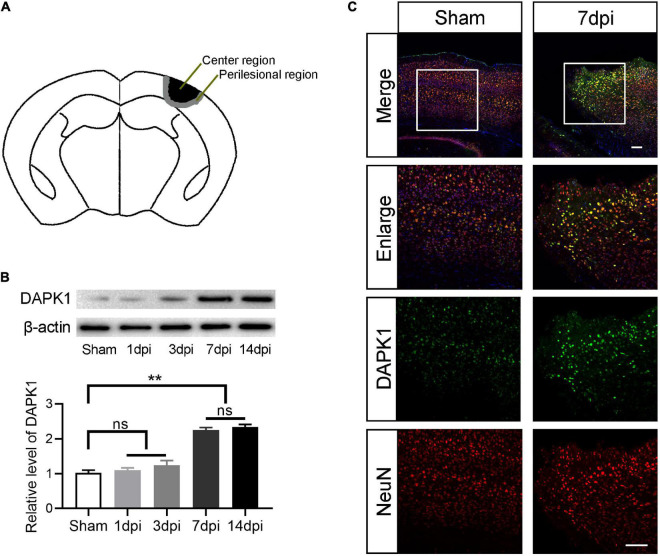
DAPK1 is increase after TBI and mainly expressed in neurons. **(A)** Perilesional of the TBI was indicated in black and center of the TBI was represented in gray. **(B)** Western blotting analysis of the expression level of DAPK1 in the perilesional cortex at different time points after TBI. **(C)** Representative immunofluorescence images in the perilesional cortex at day 7 after TBI. Scale bar, 100 μm. *n* = 6 for each group; **p < 0.01; ns, no significance; Values are presented as the mean ± SEM.

Perilesional and center cortex of the TBI are defined as shown in Figure 1A. The diameter of center cortex is 2.0 mm and the width of perilesional cortex is 0.5 mm. The expression level of DAPK1 in the perilesional cortex was examined by Western blotting at different time points after TBI. Compared to the sham group, the expression level of DAPK1 continued to rise on days 1, 3, and 7 after TBI. However, no significant increase in expression level was observed after on day 14 compared to day 7 after TBI (Figure 1B); thus, we chose day 7 after TBI for the following experiments. Furthermore, the immunofluorescence results showed that DAPK1 was mainly co-labeled with neuron rather than astrocyte or microglia (Figure 1C and Supplementary Figure 1).

### Loss of miR-124 in the Traumatic Brain Injury Mice Induces Death-Associated Protein Kinase 1 Upregulation by Post-transcriptional Regulation

**FIGURE 2:**
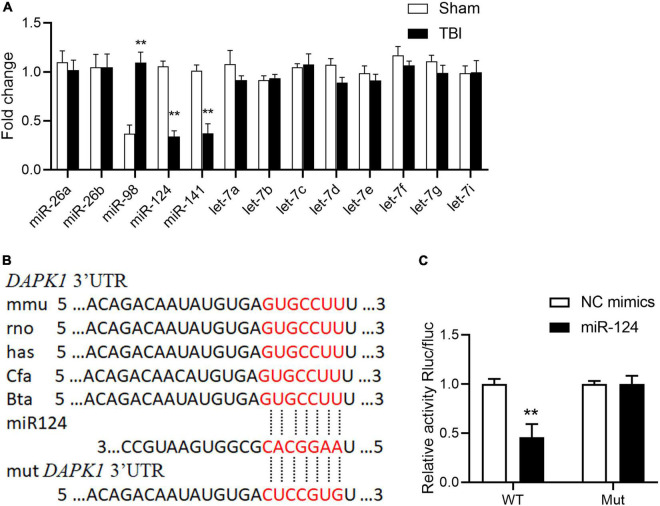
Loss of miR-124 is responsible for DAPK1 elevation in TBI. **(A)** Alterations in predicted miRNAs that target DAPK1 in the perilesional cortex of sham and TBI mice. **(B)** The binding sites of miR-124 with DAPK1 are conserved in mammalians. **(C)** The WT and Mut of DAPK1 were subcloned into the pSI-Check2 vector and transfected into HEK 293T together with miR-124 or NC mimics. The luciferase intensity was measured. *n* = 3 for each group; **p < 0.01; Values are presented as the mean ± SEM.

The mechanisms underlying DAPK1 upregulation in TBI mice were then investigated. The post-transcriptional regulation of miRNAs has recently received much attention, and we speculate that the increase in DAPK1 expression may be regulated by miRNAs. Firstly, we analyzed the 3′ untranslated regions (3′UTR) of the DAPK1 gene through miRNA.org and TargetScan 7.0; and found that miR-26a, miR-26b, miR-98, miR-124, miR-141, and let-7 family were scored the highest in both predicted outputs. The expression levels of these miRNAs in the perilesional cortex of TBI mice were determined by Reverse Transcription and quantitative Polymerase Chain Reaction (RT-qPCR). The results revealed that the expression levels of miR-98, miR-124, and miR-141 were significantly changed following TBI, whereas the expression levels of the other miRNAs remained unchanged (Figure 2A). Only miR-124 was reduced in the plasma of TBI patients with unfavorable outcome compared to patients with favorable outcome (Supplementary Figure 2). The post-transcriptional regulation of DAPK1 by miR-124 was determined using a dual-luciferase reporter assay. Our findings demonstrated that miR-124 suppressed luciferase activity in WT constructs but not in mutant constructs (Figures 2B,C). These results imply that miR-124 regulates DAPK1 expression and that loss of miR-124 causes DAPK1 upregulation in TBI mice.

### Overexpression of miR-124 or Knockdown Death-Associated Protein Kinase 1 Rescued Memory and Motor Change After Traumatic Brain Injury

**FIGURE 3:**
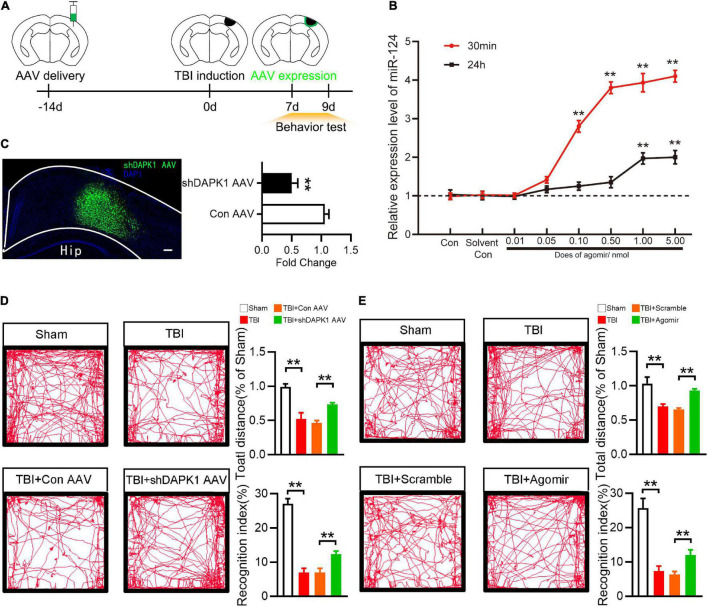
Overexpression of miR-124 or knockdown DAPK1 rescued memory and motor change after TBI. **(A)** Experimental timeline of AAV injection, TBI induction and behavior tests. **(B)** The Expression level of miR-124 in the perilesional cortex at different time points and different dose after agomir delivery. **(C)** Fluorescence image represent expression of AAV-shDAPK1 in the cortex, and quantitative analysis of the expression level of DAPK1 after AAV-shDAPK1 or AAV-Con injection. Scale bar, 100 μm. **(D)** Representative open field activity tracks and statistical analysis of total distance traveled and recognition index after AAV-shDAPK1 or AAV-Con injection. **(E)** The representative open field activity tracks and statistical analysis of total distance traveled and recognition index after agomir or scramble injection. *n* = 6 for each group; **p < 0.01; Values are presented as the mean ± SEM.

To investigate the roles of DAPK1 and miR-124 in TBI mice, agomir was used to overexpress miR-124, and AAV-shDAPK1 was used to knock down DAPK1 expression (Figure 3A). Agomir of miR-124 was delivered to the brain *via* the intranasal route in this study. Intranasal delivery has been reported to treat neurological diseases since it could effectively cross the blood-brain barrier (Lochhead and Thorne, 2012). At different doses (0, 0.01, 0.05, 0.10, 0.50, 1.00, and 5.00 nmol) and time points (30 min or 24 h after agomir was delivered) the expression level of miR-124 in the cortex was observed by RT-qPCR. The results showed that 1.00 nmol miR-124 agomir could be delivered intranasally to the cortex and achieve a 4.0-fold peak at 30 min and a 1.9-fold peak at 24 h compared to the solvent control (Figure 3B), and this does was selected as an optimum dose for the following experiments.

Similarly, shDAPK1 AAV or Con AAV were injected into the prospective injured cortex to reduce the expression level of DAPK1. AAV was injected into the brain two weeks before TBI induction and was expressed one week later (Figure 3C). In this work the damaged brain areas are largely located in sensorimotor cortex. TBI could damage hippocampal-cortex circuits and produces prominent learning and memory deficits (Paterno et al., 2017). Besides, sensorimotor cortex controls whisker movements and contributes to learned, whisker-dependent, goal-directed behaviors, which are involve in learning and object recognition (Petersen, 2019). Thus, the motor and memory behaviors of TBI mice were evaluated using an open field test and a novel object recognition test, respectively. In the open field test, TBI and TBI + Con AAV mice showed impaired ambulation compared to sham mice, whereas TBI + shDAPK1 AAV mice showed rescued ambulation compared to TBI + Con AAV mice; in the object recognition test, TBI and TBI + Con AAV mice showed a lower recognition index than the sham group, while TBI + shDAPK1 AAV mice showed a higher recognition index compared to TBI + Con AAV mice (Figure 3D). Similarly, overexpression of miR-124 in the TBI + agomir group could alleviate TBI-induced motor and memory dysfunction (Figure 3E).

### Overexpression of miR-124 or Knockdown Death-Associated Protein Kinase 1 Reduced Apoptosis and the Expression Level of Phosphorylated NR2B

**FIGURE 4:**
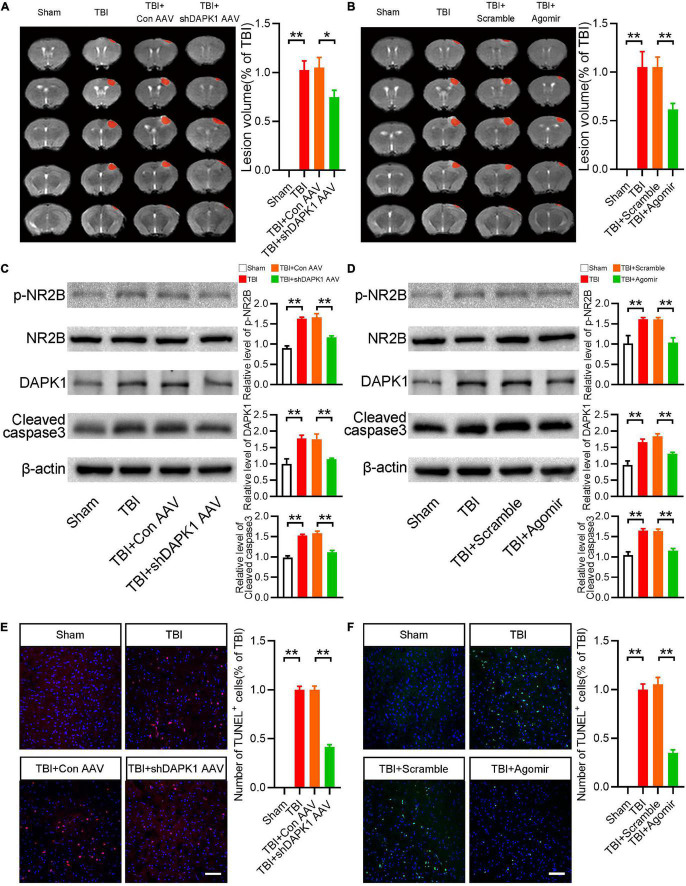
Overexpression of miR-124 or knockdown DAPK1 reduced apoptosis and the expression level of phosphorylated NR2B. **(A)** Representative MRI and statistical analysis of lesion volume after AAV-shDAPK1 or AAV-Con injection. **(B)** Representative MRI and statistical analysis of lesion volume after agomir or scramble injection. **(C)** Western blotting analysis of the expression levels of p-NR2B/NR2B, DAPK1 and cleaved caspase3 after AAV-shDAPK1 or AAV-Con injection. **(D)** Western blotting analysis of the expression levels of p-NR2B/NR2B, DAPK1 and cleaved caspase3 after agomir or scramble injection. **(E)** Representative TUNEL staining images and quantitative analyses of TUNEL^+^ cells after AAV-shDAPK1 or AAV-Con injection. **(F)** Representative TUNEL staining images and quantitative analyses of TUNEL^+^ cells after agomir or scramble injection. *n* = 6 for each group; **p < 0.01, *p < 0.05; Values are presented as the mean ± SEM.

We then studied how miR-124 and DAPK1 could influence motor and memory dysfunctions after TBI. Initially, the results in TBI mice showed that overexpression of miR-124 or knockdown of DAPK1 significantly reduced lesion volume (Figures 4A,B). Furthermore, Western blotting results revealed that delivering miR-124 agomir or knocking down DAPK1 significantly reduced TBI-induced upregulation of cleaved caspase3 (Figures 4C,D). It has been reported that DAPK1 phosphorylates NR2B in stroke (Wang et al., 2017). Whether DAPK1 phosphorylates NR2B in TBI is unknown. As a result, we examined the p-NR2B expression levels in the various groups. The expression level of p-NR2B was significantly higher in TBI and TBI + Con AVV mice than sham mice, whereas TBI + shDAPK1 AAV mice showed a lower expression level of p-NR2B compared to TBI + Con AAV mice (Figure 4C). Similarly, in the TBI + agomir group, overexpression of miR-124 could mitigate TBI-induced high p-NR2B expression (Figure 4D). Furthermore, the Western blotting results confirmed the downregulation of DAPK1 in mice after agomir or shDAPK1 AAV injection (Figures 4C,D). TUNEL staining results showed that decreased apoptosis level in TBI mice after agomir or shDAPK1 AAV injection (Figures 4E,F).

### Tat-NR2B Could Alleviate Motor and Memory Dysfunction of Traumatic Brain Injury Mice and Reduce Apoptosis and the Expression Level of Phosphorylated NR2B

**FIGURE 5:**
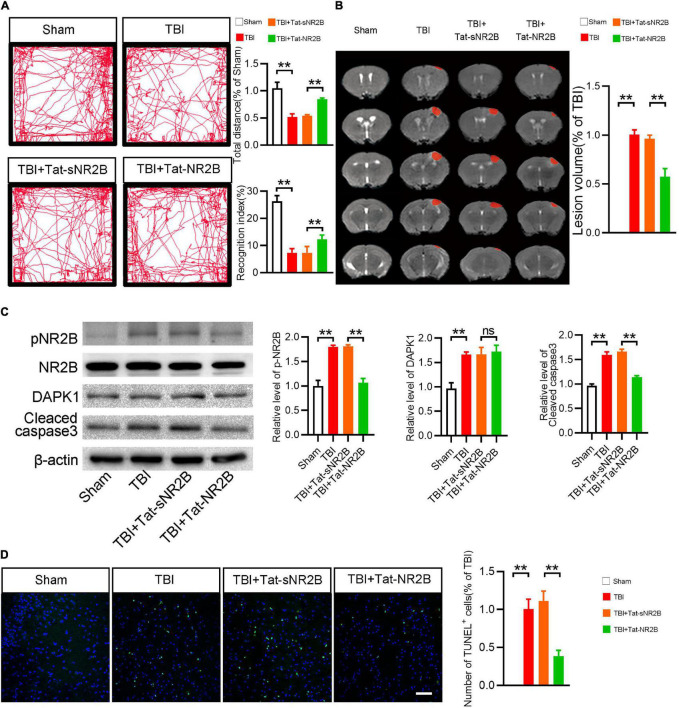
Tat-NR2B could alleviate motor and memory dysfunction of TBI mice and reduce apoptosis and the expression level of phosphorylated NR2B. **(A)** Representative open field activity tracks and statistical analysis of total distance traveled and recognition index in each group. **(B)** Representative MRI and statistical analysis of lesion volume in different groups. **(C)** Western blotting analysis of the expression levels of p-NR2B/NR2B, DAPK1 and cleaved caspase3. **(D)** Representative TUNEL staining images and quantitative analyses of TUNEL^+^ cells in each group. *n* = 6 for each group; **p < 0.01; ns, no significance; Values are presented as the mean ± SEM.

In this study, a transmembrane peptide called Tat-NR2B was used to specifically inhibit the binding of DAPK1 and NR2B (Tu et al., 2010). Behavioral and pathological changes of the mice were observed, as mentioned before. The TBI mice showed rescued ambulation and higher recognition index after Tat-NR2B injection (Figure 5A). Lesion volume was significantly reduced after Tat-NR2B injection (Figure 5B). Western blotting results showed that the expression level of cleaved caspase3 was decreased considerably in TBI + Tat-NR2B group compared to TBI + Tat-sNR2B group. Furthermore, after the Tat-NR2B injection, p-NR2B/NR2B expression was significantly reduced in TBI mice, whereas DAPK1 remained unchanged (Figure 5C). TUNEL staining results showed decreased apoptosis level in perilesional cortex after Tat-NR2B injection (Figure 5D). These findings suggested that DAPK1/NR2B pathways involved neuronal apoptosis following TBI.

### Tat-NR2B Didn’t Affect the Number and Morphology of Synapse

**FIGURE 6:**
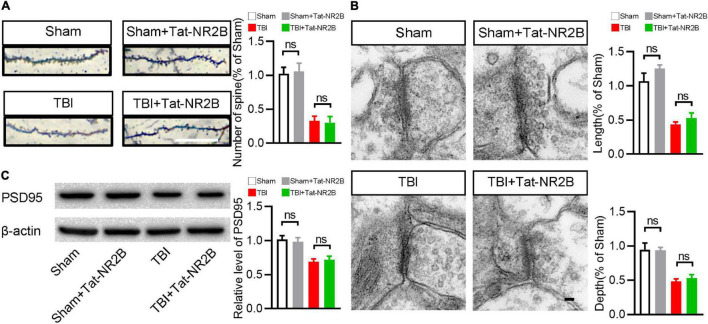
Tat-NR2B didn’t affect the number and morphology of synapse. **(A)** Representative Golgi staining images and statistical analysis of dendritic spine number. Scale bar, 10 μm. **(B)** Representative transmission electron microscope images and statistical analysis of length and depth of synapse. Scale bar, 100 nm. **(C)** Western blotting analysis of the expression levels of PSD95. *n* = 6 for each group; ns, no significance; Values are presented as the mean ± SEM.

In addition to participating in neuronal apoptosis, NR2B is also related to synapse structures and functions. It is unclear whether specifically blocking the bind between DAPK1 and NR2B affects the number and morphology of synapses. Golgi staining revealed no significant difference in the number of synapses after Tat-NR2B injection in TBI or sham mice (Figure 6A). Transmission electron microscopy results revealed that the depth and length of synapse in TBI or sham mice remained unchanged after Tat-NR2B injection (Figure 6B). Furthermore, Western blotting results showed that the expression level of PSD95 was unchanged after Tat-NR2B injection in TBI or sham mice (Figure 6C). These results showed that inhibiting the relationship between DAPK1 and NR2B did not affect the number or morphology of synapses.

## Discussion

This study reported that the expression level of DAPK1 is upregulated after TBI, which is positively correlated with poor prognosis in TBI patients. We demonstrated that the loss of miR-124 caused DAPK1 upregulation. Then, we found that neuronal apoptosis and NR2B phosphorylation after TBI could be alleviated by knockdown DAPK1 expression in the cortex, which further moderated motor and memory impairment in mice. Finally, neuronal apoptosis and neurological function were improvedby administering Tat-NR2B, specifically blocking DAPK1 binds to NR2B. Golgi staining, Western blotting and transmission electron microscopy showed that the number and morphology of synapse were not affected by Tat-NR2B.

DAPK1 has been shown to play a role in various neurological disorders. Current TBI research has revealed a link between Tau and DAPK1 in the chronic phase (Kim et al., 2021). However, whether and how DAPK1 regulates neuron death in the acute phase is still unknown. We have observed a significant increase of DAPK1 in TBI patients’ plasma with unfavorable outcome and in the perilesional cortex of the mice, and DAPK1 is mainly expressed in the neurons. These findings suggested that DAPK1 may play an essential role in neuronal apoptosis of TBI mice.

To begin, we investigate the mechanism of DAPK1 upregulation. miRNA is a short non-coding RNA that binds to mRNA specifically for post-transcriptional regulation (Bartel, 2009). miRNA has been reported to play a role in various neurological disorders (Mehta et al., 2020). For example, In AD, stroke, and TBI, the relationship between neuroprotection and miR-124 has been reported (Wang et al., 2018; Liu et al., 2019; Yang et al., 2019). Clinical research has reported downregulation of miR-124 in the dentate gyrus of TBI patients (Schindler et al., 2020). The primary analysis of miR-124 in TBI models mainly focused on neuronal inflammation and microglia polarization (Huang et al., 2018; Yang et al., 2019). It is unknown whether miR-124 has any other downstream mechanisms in TBI. Our findings confirmed the downregulation of miR-124 in TBI mouse brain tissue and TBI patient plasma. The dual-luciferase assay showed that miR-124 could specifically bind to DAPK1, inhibit the translation of DAPK1 and reduce the expression level of DAPK1 protein. These results showed that DAPK1 is one of the downstream targets of miR-124. We infer that the effect of agomir could be blocked by knocking down DAPK1 prior to the administration of agomir, and this possibility should be addressed in further research.

Agomir is a chemically modified miRNA (Henshall et al., 2016); *in vivo* injection of agomir can significantly increase miR-124 expression and cause pathological and behavioral changes in TBI mice. Intranasal delivery may be a potential route of administration in the future because it is non-invasive and can cross the blood-brain barrier (Erdo et al., 2018). In AD mouse, intranasal administration of miR-146a agomir promotes the pathological process and cognitive impairment (Mai et al., 2019). Furthermore, intranasal delivery of miR-219 agomir is considered a potential target for treating Theiler’s Virus-Induced Demyelinating Disease (Moyano et al., 2018).

The expression of DAPK1 was reduced by injecting AAV-shDAPK1 into the cortex. Later, behavioral tests revealed that overexpression of miR-124 or knockdown of DAPK1 significantly improved TBI mice’s motor and memory functions. DAPK1 is the primary key protein kinase that regulates cell death in the brain in various neurodegenerative diseases such as PD and AD (Nair et al., 2013; Kim et al., 2019). To our knowledge, this is the first study to show that DAPK1 can be used to treat TBI-induced cell death in mice.

Further pathological studies revealed that overexpression of miR-124 or knockdown of DAPK1 could reduce lesion volume and the level of apoptosis. Earlier studies have shown that the apoptotic mechanism contributes to the overall pathology of TBI, and the excessive activation of the apoptotic mechanismmay be harmful (Itoh et al., 2013; Akamatsu and Hanafy, 2020). In AD and stroke, DAPK1 is linked to apoptosis (Kim et al., 2019), and these findings show that DAPK1 alsoplays a protective role in TBI by inhibiting the apoptosis of neurons.

The interaction between NR2B and DAPK1 in stroke has been reported (Tu et al., 2010). The expression of NR2B Ser1303 was reduced after overexpression of miR-124 or knockdown of DAPK1, indicating that DAPK1 may be involved in the phosphorylation of NR2B. NR2B is a regulator of Ca^2+^ channels and is closely related to apoptosis and synapse function (Martel et al., 2009). In this study, we are the first to demonstrate the relation between apoptosis and DAPK1/NR2B in TBI. Phosphorylating NR2B could directly resulting in Ca^2+^ overload; besides, the NR2B–PSD95 complex interaction with CaMK II, could indirectly resulting in excessive Ca^2+^ influx (Irie et al., 1997). Ca^2+^ overload results in excitotoxicity, endoplasmic reticulum stress and mitochondrial dysfunction, which ultimately induced expression of cleaved caspase-3 (Orrenius et al., 2003). In ischemia reperfusion, DAPK1-ERK signal contributing to neuronal apoptosis (Xiong et al., 2018); while in this study the expression level of p-ERK1/2 was not changed in TBI group nor by any of the rescue strategies (Supplementary Figure 3), we infer the discordance could be ascribed to various disease models and disparate detection time point.

To demonstrate the role of DAPK1 in the phosphorylation of NR2B in TBI mice, we used Tat-NR2B to block the combination of DAPK1 and NR2B specifically. TBI mice’s motor and memory functions were restored after intraperitoneal injection of Tat-NR2B. TBI mice showed a significant decrease in lesion volume and level of apoptosis. These findings suggested that DAPK1 plays an essential role in apoptosis following TBI *via* NR2B. Inhibiting the binding of DAPK1 and NR2B has reduced neuronal apoptosis and restored neuronal functions.

Transcriptional activator protein (Tat) is a transmembrane peptide and has been widely used in drug delivery for diseases because of its low toxicity and high safety (Zou et al., 2013). In animal studies, Tat has been used to bind with multiple neuroprotective proteins to reduce neuron degeneration in PD and protect neurons against cerebral ischemic insults (Tu et al., 2010; Su et al., 2019). Furthermore, Tat-mediated drug delivery has been used in clinical trials and is undergoing phase II trials (Guidotti et al., 2017). Tat peptide could be a future target for the treatment of TBI dysfunction.

Aside from its role in apoptosis, NR2B also plays an essential role in mammalian synapse maturation (Frank et al., 2016); moreover, a rare variant discovered within NR2B C-Terminus in autistic patients affects NMDA receptor surface expression and synapse density (Liu et al., 2017). Furthermore, the stability of NR2B in the nucleus accumbens regulates behavioral and synaptic adaptations to chronic stress (Jiang et al., 2013). It is still unknown whether inhibiting the DAPK1/NR2B pathway affects synaptic number and morphology in TBI mice. In previous study, electrophysiological patch-clamp recording showed that uncoupling of an activated DAPK1 from the NMDA receptor complex protects against brain damage in stroke without affecting the physiological actions of the NMDA receptors (Tu et al., 2010); our results show that after the interaction between DAPK1 and NR2B was blocked by Tat-NR2B, the number and morphology of synapse did not change, which were consistent with above electrophysiological results. We speculated that the phenomenon might be related to the unchanged PSD95 level. PSD95 is a critical synaptic protein that binds to the NMDA receptors and controls synaptic transmission and plasticity (Beique et al., 2006), while overexpression of PSD-95 increases synaptic transmission and blocks long-term depression (Xu et al., 2008; Dore and Malinow, 2021). These findings imply that Tat-NR2B can only influence neuronal apoptosis without affecting number and morphology of synapse, which support the safety of blocking DAPK1/NR2B pathways in TBI treatment to some extent. However, the electrophysiological validation of these findings is lacking, further in electrophysiological patch-clamp recording experiments are awaited to validate these findings in the future.

Overall, our findings indicate that the loss of miR-124 following TBI is associated with increased DAPK1 expression. Inhibiting DAPK1 expression could reduce NR2B phosphorylation, decrease apoptosis in the perilesional cortex, and improve memory and motor behavioral tests in TBI mice. These studies provide a target for the treatment of TBI-related dysfunction.

## Data Availability Statement

The raw data supporting the conclusions of this article will be made available by the authors, without undue reservation.

## Ethics Statement

The studies involving human participants were reviewed and approved by the Ethics Committee of Tangdu Hospital, Fourth Military Medical University. The patients/participants provided their written informed consent to participate in this study. The animal study was reviewed and approved by the Ethics Committee of Tangdu Hospital, Fourth Military Medical University.

## Author Contributions

YQ and HL developed the hypotheses and designed the experiments. YS, WC, and QW performed the experiments and wrote the manuscript. JZ and XW contributed to the collection and analysis of patient samples. JW and SZ helped to draft the manuscript. QH and LH analyzed the data. YD and SG collected the clinical data. All authors contributed to the article and approved the submitted version.

## Conflict of Interest

The authors declare that the research was conducted in the absence of any commercial or financial relationships that could be construed as a potential conflict of interest.

## Publisher’s Note

All claims expressed in this article are solely those of the authors and do not necessarily represent those of their affiliated organizations, or those of the publisher, the editors and the reviewers. Any product that may be evaluated in this article, or claim that may be made by its manufacturer, is not guaranteed or endorsed by the publisher.

## Supplementary Material

Supplementary Figure 1 Expression pattern of DAPK1 in the cortex; Scale bar, 100 μm.

Supplementary Figure 2 Alterations of miR-98, miR-124, and miR-141 in the blood plasma of TBI patients.

Supplementary Figure 3 Expression level of p-ERK/ERK in different groups.
