# Supplementary figures and images for: MicroRNA-124/Death-Associated Protein Kinase 1 Signaling Regulates Neuronal Apoptosis in Traumatic Brain Injury via Phosphorylating NR2B
Source: Front Cell Neurosci. 2022 Jun 15;16:892197. doi: 10.3389/fncel.2022.892197 (PMC9240278; doi:10.3389/fncel.2022.892197)

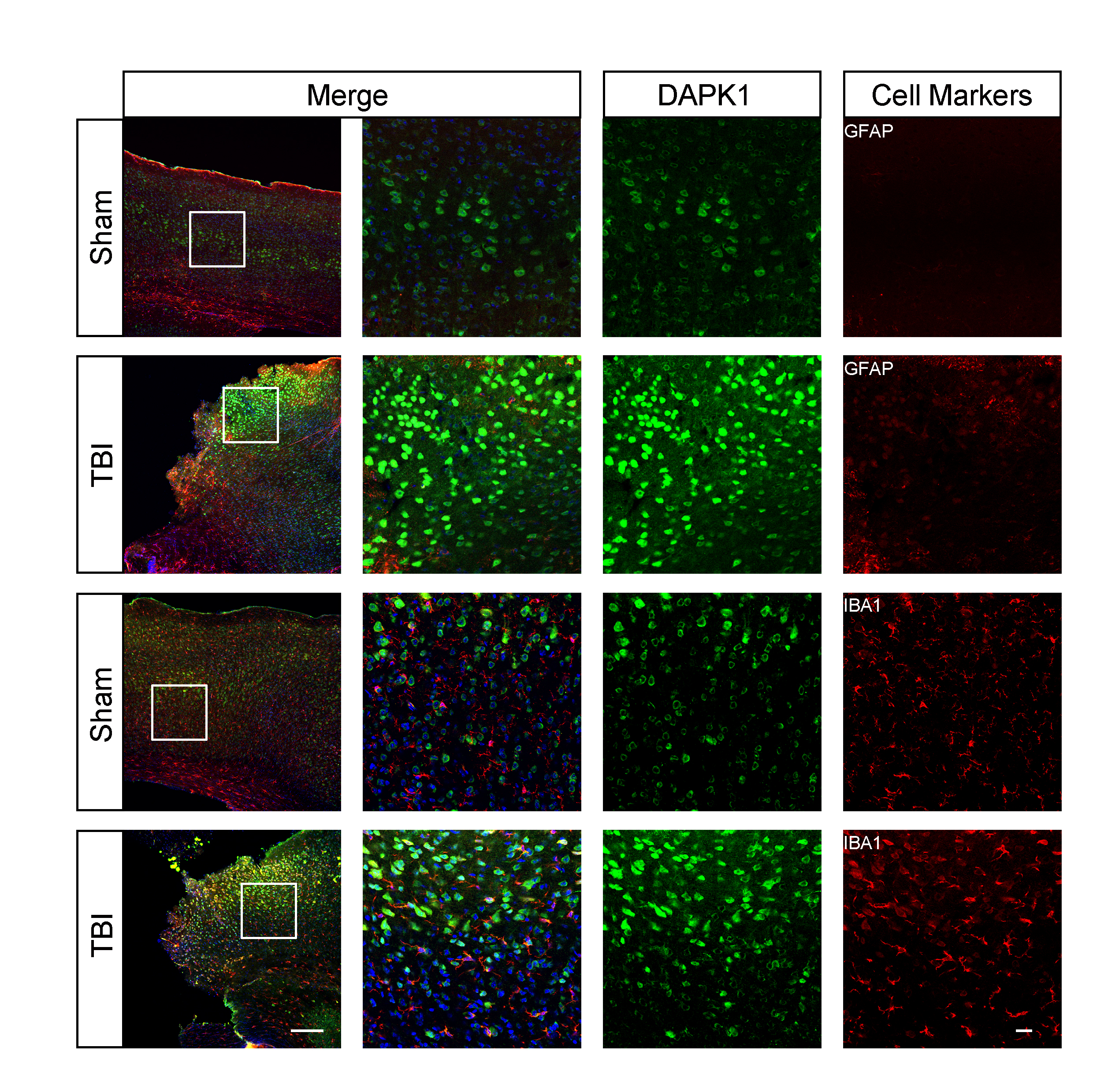

Supplement: Supplementary Figure 1 — Expression pattern of DAPK1 in the cortex; Scale bar, 100 μm. [file Image_1.JPEG]

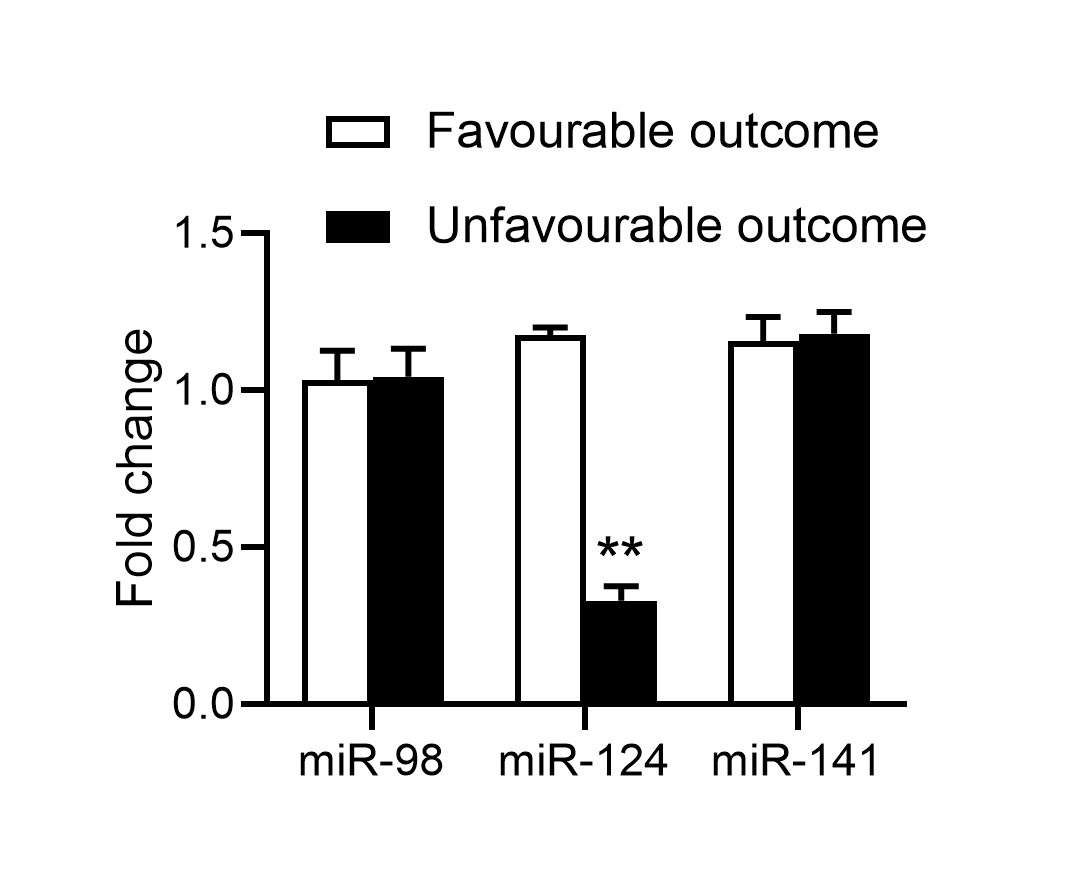

Supplement: Supplementary Figure 2 — Alterations of miR-98, miR-124, and miR-141 in the blood plasma of TBI patients. [file Image_2.JPEG]

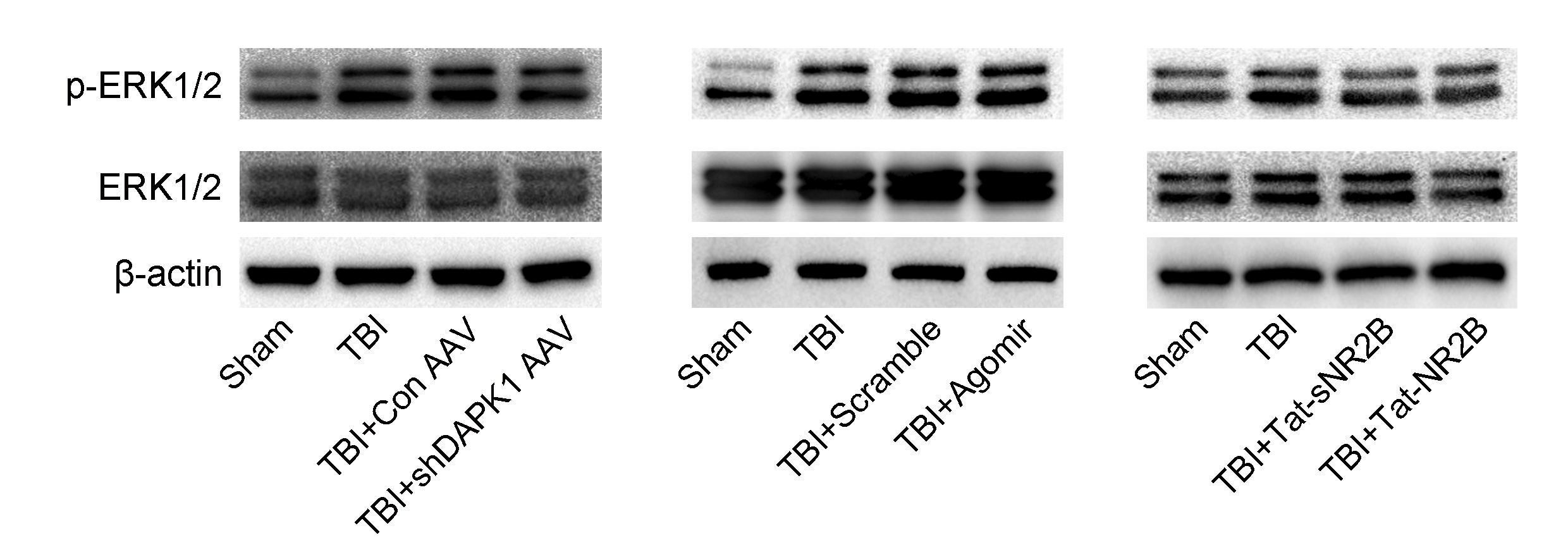

Supplement: Supplementary Figure 3 — Expression level of p-ERK/ERK in different groups. [file Image_3.JPEG]
